# Effects of Nicotine-Free E-Cigarettes on Gastrointestinal System: A Systematic Review

**DOI:** 10.3390/biomedicines13081998

**Published:** 2025-08-16

**Authors:** Ivana Jukic, Ivona Matulic, Jonatan Vukovic

**Affiliations:** 1 Department of Internal Medicine, Division of Gastroenterology, University Hospital of Split, Spinciceva 1, 21000 Split, Croatia; jonatan.vukovic@mefst.hr; 2 Faculty of Health Sciences, University of Split, Ulica Rudjera Boskovica 35, 21000 Split, Croatia; 3 Private Clinic Matulic, Osjecka Ulica 24a, 21000 Split, Croatia; 4 Department of Internal Medicine, School of Medicine, University of Split, Soltanska 2, 21000 Split, Croatia

**Keywords:** nicotine-free e-cigarettes, gastrointestinal health, gastrointestinal system, systematic review, tobacco-free e-cigarettes, zero-nicotine e-cigarettes

## Abstract

**Background/Objectives:** Nicotine-free electronic cigarettes (NFECs) are becoming increasingly popular, especially among youth and non-smokers, yet their effects on the gastrointestinal tract (GIT) remain poorly understood. This systematic review synthesizes available in vitro, in vivo, and limited human evidence on NFEC-associated changes in gastrointestinal health and function. **Methods:** Literature searches were conducted in Medline, Web of Science, Cochrane, and Scopus in July 2025, following PRISMA guidelines. Eligible studies examined NFEC effects on any GIT segment, including the oral cavity, liver, intestines, and microbiome. Data on study design, exposure characteristics, and main outcomes were extracted and narratively synthesized. **Results:** Of 111 identified records, 94 full-text articles were retrieved, and 21 studies met the inclusion criteria. Most were preclinical, with only one human pilot study. Evidence from oral cell and microbial models suggests that NFEC aerosols can induce pro-inflammatory cytokine production, impair cell viability, and disrupt microbial metabolism through their base constituents (propylene glycol, vegetable glycerine, and flavourings). Animal studies indicate possible hepatic oxidative stress, altered lipid metabolism, and gut barrier dysfunction, with some data suggesting more pronounced steatosis in nicotine-free exposures compared to nicotine-containing counterparts. Microbiome studies report reduced tight junction expression and altered neutrophil function. **Conclusions:** Current evidence is limited and predominantly preclinical but indicates that NFEC exposure can affect multiple aspects of gastrointestinal health. Robust longitudinal and interventional human studies are urgently needed to determine the clinical relevance of these findings and to inform regulation and public health policy.

## 1. Introduction

Cigarette smoking remains a leading global cause of preventable morbidity and mortality and is responsible for over 8 million deaths annually, primarily due to its established links with cardiovascular disease, chronic respiratory conditions, and multiple cancers [1,2,3,4]. In response to tobacco control efforts and increasing public health awareness, the past two decades have seen a sharp rise in the popularity of electronic cigarettes (e-cigarettes, ECs), marketed as a safer alternative to combustible tobacco and a smoking cessation aid [5,6,7]. The concept of e-cigarettes dates back to the early 1960s, following growing recognition of tobacco’s toxic effects. One example is the British American Tobacco-initiated “Project Ariel,” an early attempt to develop a smokeless electronic device aimed at delivering nicotine while filtering out harmful combustion products [8]. Gilbert patented a “Smokeless Non-Tobacco Cigarette,” proposing vaporization of flavoured liquids without combustion in 1962 [9]. However, these early projects were abandoned due to immature technology, unfavourable market conditions, and industry reluctance.

Modern ECs were developed in 2003 by Chinese pharmacist Hon Lik and introduced commercially in 2004 [10], based on the vaporization of an e-liquid base constituent—most commonly composed of propylene glycol (PG) and vegetable glycerine (VG) as the primary base solvents for nicotine and flavourings—using a battery-powered heating coil [11,12].

Global EC use has since expanded dramatically: by 2021, the global vaping market was valued at over USD 18 billion, with more than 400 different brands of e-cigarettes available, and usage among adolescents and young adults outpaced that of older populations in many regions [13,14,15,16].

While much attention has focused on nicotine-containing e-cigarettes, a growing number of users, particularly youth and never-smokers, now consume nicotine-free e-cigarettes (NFECs) [17,18]. Marketing these products through various platforms that are easily accessible to youth further drives the perception of e-cigarette smoking as a harmless trend [19,20,21,22]. Furthermore, dual use of both traditional and electronic cigarettes is increasing, and electronic cigarettes are increasingly used more as a complementary rather than a substitutional habit to traditional tobacco smoking [23,24].

Regulatory oversight of e-cigarettes varies widely. The U.S. Food and Drug Administration (FDA) regulates electronic nicotine delivery systems (ENDSs) under the Tobacco Control Act, but enforcement for nicotine-free devices remains inconsistent [25,26,27,28]. In the European Union, the Tobacco Products Directive excludes non-nicotine liquids, leaving gaps in standardization. In 2015, the European Commission implemented two acts and a commission report related to e-cigarettes, establishing a common format for e-cigarette labelling, technical standards for e-cigarette refilling mechanisms, and their current established risks to public health [29]. Many countries have prohibited the sale of nicotine e-cigarettes to minors but permit or inadequately regulate nicotine-free variants, most commonly through flavour restrictions [30,31,32]. This lack of a regulatory patchwork complicates safety evaluation, consumer awareness, and research harmonization [33,34].

Despite their inhalational route, e-cigarette vapours interact with the gastrointestinal tract (GIT) in several ways: via direct contact with the oral mucosa, swallowing aerosol condensates, systemic absorption of volatile compounds, and hepatic metabolism. The GIT, with its immunological, epithelial, and microbial complexity, may be particularly vulnerable to such exposures. Several studies have shown e-cigarette vapours to exert various biological effects, even in the absence of nicotine [35,36,37,38].

Given the rapid growth in NFEC usage, a comprehensive review of the effects of NFECs on the gastrointestinal system is both timely and necessary. This systematic review aims to evaluate available evidence on the impact of nicotine-free e-cigarette exposure on the gastrointestinal tract, defined broadly from the oral cavity to the rectum, including the liver, pancreas, and gut–brain axis.

## 2. Materials and Methods

### 2.1. Information Source and Search Strategies

This systematic review was conducted in accordance with the *Cochrane Handbook* and adhered to Preferred Reporting Items for Systematic Review and Meta-Analyses (PRISMA) guidelines to ensure appropriate quality of the assessment. This review was registered on the Open Science Framework (OFS) (https://doi.org/10.17605/OSF.IO/MXNY8). The protocol outlines the objectives, search strategy, and inclusion criteria for evaluating the effects of nicotine-free e-cigarettes on the gastrointestinal system.

A comprehensive literature search was conducted in July 2025 via the Medline, Web of Science, Cochrane, and Scopus electronic databases to identify relevant studies exploring the gastrointestinal effects of nicotine-free electronic cigarette vapour exposure.

In vitro and in vivo studies of the effects of nicotine-free e-cigarette exposure were included.

Both free-text terms and MeSH-indexed terms were used in combination, with Boolean operators (AND, OR) applied to maximize the retrieval of desired reports. Example search strings included “nicotine-zero e-cigarette” OR “nicotine-free e-cigarette” AND “gastrointestinal health”.

The search aimed to capture all studies, without language barriers, addressing in vitro, in vivo (animal, human, microbiological), and clinical aspects of nicotine-free e-cigarette use and the effects of PG, VG, and flavouring agents in the context of gastrointestinal function. Reference lists of selected articles were manually screened to identify additional relevant publications. Duplicate records were removed. Duplicate records were identified by matching the title, author list, year of publication, journal name, and DOI. Records meeting all these criteria were considered duplicates, and only one copy was retained for screening. The screening and selection process was independently conducted by three authors (I.J., J.V., I.M.). In cases where initial inclusion or exclusion decisions were not unanimous, disagreements were discussed collectively until consensus was reached. If consensus could not be achieved, the decision was made by majority vote. First, titles and abstracts were evaluated for relevance, and full texts of potentially eligible studies were obtained and reviewed in detail to determine final inclusion based on predefined criteria. From each eligible study, we extracted information on the study design, applied model (in vitro, in vivo, microbiological, clinical), sample characteristics, exposure details (including PG/VG ratio and flavourings when reported), measured outcomes, and key findings. Data extraction was performed independently by all three reviewers (I.J., I.M., J.V.), and any discrepancies were resolved through discussion until consensus was reached. A narrative synthesis was performed to summarize findings across different studies due to the heterogeneity of available data. Data were organized by gastrointestinal tract segments and presented as both descriptive text and structured tables with a focus on key findings and gastrointestinal health implications.

The complete search strategies for each database, including all search terms, Boolean operators, and applied filters, are provided in Supplementary File S1.

This review did not include searches of grey literature sources, preprint servers, or conference abstracts. While the database search strategy was designed to capture the widest possible range of published evidence, we acknowledge that relevant emerging studies on NFECs—particularly given the rapid pace of e-cigarette research—may exist outside the peer-reviewed literature.

### 2.2. Eligibility Criteria

This systematic review reviewed all in vitro and in vivo available studies that investigated the effects of nicotine-free e-cigarette liquids on the gastrointestinal system.

Inclusion criteria for this review were as follows: (1) peer-reviewed original research articles without language barriers; (2) studies evaluating the effects of nicotine-free e-cigarettes or isolated PG/VG on the gastrointestinal tract; (3) in vitro studies using gastrointestinal cell models; (4) in vivo studies in animals or humans assessing gastrointestinal outcomes; (5) research on e-liquid pharmacokinetics, metabolism, and excretion; (6) studies involving paediatric, adolescent, or adult populations.

Exclusion criteria were as follows: (1) studies focusing solely on nicotine-containing e-cigarettes; (2) articles not related to gastrointestinal endpoints; (3) narrative reviews, editorials, or commentaries; (5) articles without full-text availability; and (6) duplicated publications or non-original data (Table 1).

## 3. Results

**Figure 1:**
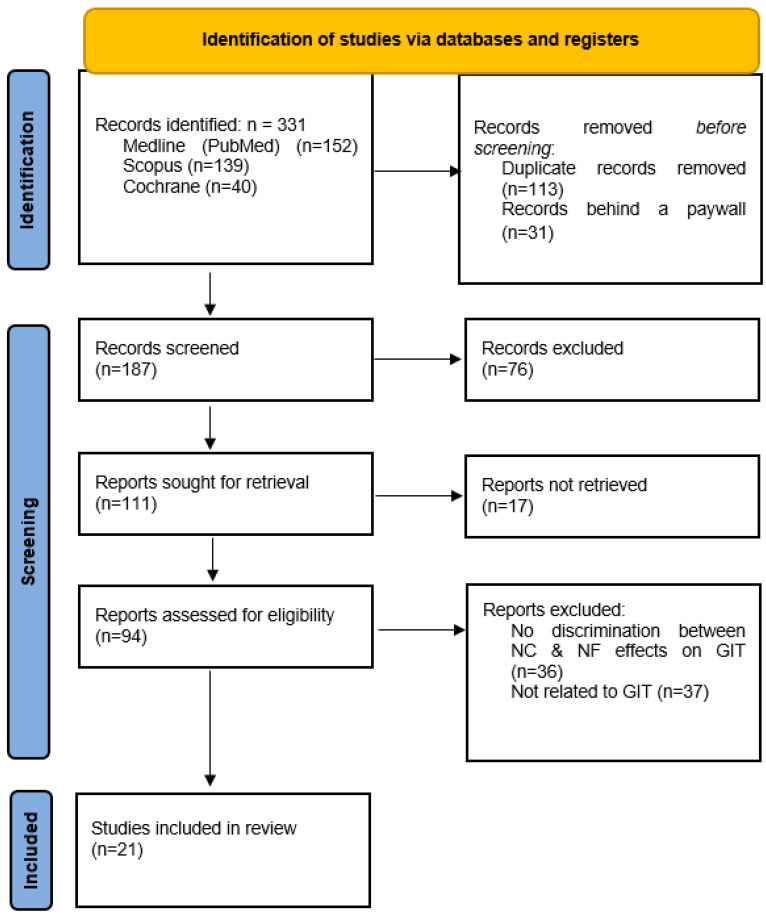
PRISMA flow diagram of database search and selection. Legend: GIT—gastrointestinal tract; NC—nicotine-containing; NF—nicotine-free.

A flow diagram of the database search and selection process is presented in Figure 1. We identified a total of 111 studies potentially eligible for this review, out of which 94 were successfully retrieved. Following further inspection, 36 studies were excluded because they did not distinguish between nicotine-free (NF) and nicotine-containing (NC) e-cigarette effects. An additional 37 studies were excluded as they did not explore the GIT effects of NFECs. Finally, a total of 21 studies met the full inclusion criteria for this systematic review [39,40,41,42,43,44,45,46,47,48,49,50,51,52,53,54,55,56,57,58,59].

### 3.1. Nicotine-Free E-Cigarettes and Oral Health

Table 2 summarizes the characteristics of current available studies on oral health in the context of NFEC exposure [39,40,41,42,43,44,45,46,47,48,49,50,51].

Nine in vitro studies of human oral cell lines observed the effects of EC exposure with regard to nicotine content [39,40,41,42,43,44,45,46,49]. A study carried out by Yu et al. in 2016 [39] investigated the cytotoxic potential of EC condensate exposure in human oral keratinocytes and head and neck squamous cell carcinoma (HNSCC) lines via several different assays and control exposure to determine nicotine-independent effects of EC exposure. They reported similar levels of DNA strand breaks, apoptosis, and decreased viability in both cell lines, as well as increased HNSCC resistance to chemotherapeutic treatment [39]. Manyanga et al. studied the chemotherapeutic resistance of HNSCC to platinum-based treatment and reported similar results [46]. Sancilio et al. contributed two studies in consideration of the effects of NFEC exposure on human gingival fibroblasts and reported similar rates of geno- and cytotoxicity in both NC and NF groups in both studies [40,41].

NFECs also impaired wound healing and cell migration. Alanzi et al. (2018) observed reduced fibroblast proliferation and delayed wound closure [42], while Rouabhia et al. (2019) reported reduced adhesion and mineralization of osteoblasts on titanium implants, suggesting a potential negative impact on dental implant integration [43]. A study performed by Tsai et al. in 2020 [44] sought to investigate how different flavours may interact with human oral cell lines and found a spectrum of effects. Some flavours were correlated with decreased neoplastic invasion, whereas others enhanced it. Beklen et al. (2021) [45] examined the effects of unflavoured nicotine-free e-liquid base constituents, PG and VG, on gingival epithelial cells. Increased cytotoxicity was found in all groups, but it was most pronounced with ECs containing more PG than VG. In vivo data were limited. Tolba et al. (2023) observed epithelial changes and taste bud damage in rats injected with NFEC liquids, although the non-physiological exposure route limits generalizability [48]. Ma et al. (2024) confirmed NFEC-induced cytotoxicity and DNA damage in oral keratinocytes, with effects comparable to acrolein exposure [49]. A rare pilot human study by Vamos et al. (2024) found no significant changes in palatal blood flow following acute exposure to NFECs, although long-term effects were not assessed [50].

Two microbiological studies were included. A study carried out by Haghighi et al. (2022) on *C. albicans* found that while nicotine enhanced *C. albicans* virulence and biofilm formation, NFECs had a minimal effect, suggesting that nicotine plays a more prominent role in fungal pathogenicity [47]. Lee-Scot Beverly et al. (2025) showed that NFEC aerosols are metabolized by the oral microbiome, with alterations in microbial metabolism, gene expression, and biofilm structure in human saliva samples [51].

### 3.2. NFEC and Liver Health and Function

Table 3 summarizes the current evidence regarding the impact of NFECs and liver health and function [52,53,54,55,56,57]. Five in vivo studies performed on rodent models demonstrated that NFECs may alter hepatic metabolism. Golli et al. compared the effects of both NC and NFEC exposure, as well as nicotine alone, on hepatic parameters, lipid peroxidation, and antioxidant activity [52]. They found altered hepatic metabolism, as evidenced by increases in liver function test (LFT) probes, enzyme activity, and lipid peroxidation in all groups, which were more pronounced in EC groups than those with nicotine alone. Nicotine presence in ECs worsened the histopathological outcomes. Chen et al. and Li et al. performed studies on dams and their offspring to identify how EC exposure may affect metabolism during and after pregnancy [53,54]. Both studies found signs of altered lipid metabolism, increased body fat percentage, and liver steatosis. Li et al. compared the effects with and without nicotine and found more pronounced liver steatosis in the NF group compared to the NC group, suggesting a potential protective effect of nicotine in counteracting liver steatosis induced by EC exposure. Rickard et al. performed a study on hepatocellular carcinoma G2 cells (HepG2) to identify the effects of different common flavourings found in EC liquids, as well as their base constituents [55]. They found chronic exposure to EC liquids decreased HepG2 cell viability. Chen et al. performed additional studies to compare the effects of a high-fat diet (HFD) to EC exposure and concluded EC exposure may induce more potent changes, promoting systemic inflammation, particularly in the liver [56].

### 3.3. NFEC and Gastrointestinal Microbiome

Table 4 presents the current available data on the effects of NCEFs on the gastrointestinal microbiome [58,59]. Two studies were identified as relevant for this review. Sharma et al. performed an in vitro study using murine and human enteroid-derived cells to examine the effects of EC use on the gut barrier. They found that chronic NFEC exposure may promote inflammation and reduce tight junction expression. Co-culture of these cell lines with bacteria showed signs of barrier disruption. A study carried out on human neutrophils by Jasper et al. showed EC exposure may significantly alter neutrophil function, reducing their phagocytic potential as evidenced by their reduced phagocytosis of *E. coli* and promoting excessive filamentous actin polymerization [59].

### 3.4. Nicotine-Free E-Cigarettes and Implications for Gastrointestinal Health

**Figure 2:**
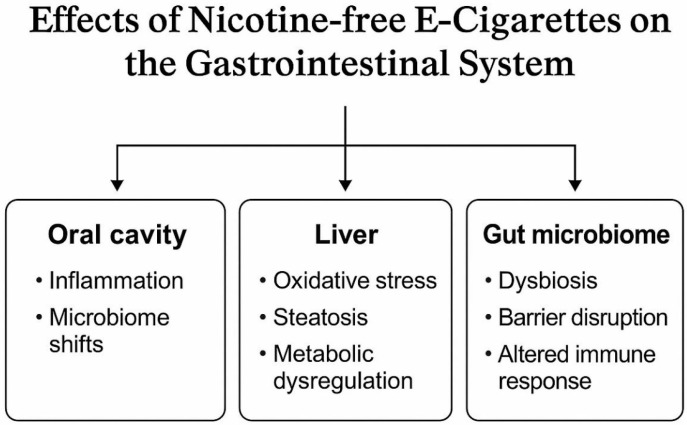
Effects of NFECs on the gastrointestinal system.

Figure 2 visually represents the effects implied in the studies included in this review. While most findings are based on in vitro or animal models, the consistency of harmful cellular responses across organ systems raises concerns about the long-term safety of NFECs. These results underscore the need for more human studies and mechanistic investigations to clarify dose–response relationships, exposure thresholds, and the role of specific e-liquid constituents in gastrointestinal toxicity.

## 4. Discussion

This systematic review provides a comprehensive synthesis of the existing evidence on the effects of nicotine-free electronic cigarette (NFEC) exposure on the gastrointestinal tract (GIT), encompassing oral health, microbial homeostasis, epithelial function, and metabolic processes. While the current body of literature remains limited, the findings suggest that NFECs are not biologically inert and may exert adverse effects on multiple levels of gastrointestinal function, even in the absence of nicotine.

The most consistent evidence emerged in relation to the oral cavity, which is the GIT region most directly exposed to EC aerosols. Several in vitro studies demonstrated that exposure to NFEC vapour leads to increased production of inflammatory mediators (IL-6, IL-8), reduced cell viability, and impaired intercellular signalling in gingival and epithelial cells. These effects were comparable to those observed with nicotine-containing e-cigarettes, supporting the hypothesis that e-liquid base constituents such as propylene glycol, vegetable glycerine, and flavouring agents may independently contribute to mucosal irritation and cytotoxicity. Notably, these effects were observed even with unflavoured formulations, suggesting that base solvents alone may have pro-inflammatory potential.

Alterations in oral microbiota were also observed, with in vitro and ex vivo studies reporting increased biofilm formation, shifts in bacterial metabolic activity, and changes in gene expression linked to xenobiotic degradation and quorum sensing. These microbial changes may have downstream implications for oral and systemic health, including enhanced risk of caries, gingivitis, and disruption of host–microbiome homeostasis. Such dysbiosis could extend beyond the oral cavity, potentially affecting the entire gastrointestinal ecosystem through microbial migration or inflammatory signalling.

The evidence regarding the effects of NFECs on the liver and intestinal segments of the GIT remains sparse and heterogeneous. Preliminary in vivo animal studies have indicated potential hepatic enzyme induction, oxidative stress, and metabolic dysregulation following chronic NFEC exposure. However, inconsistencies in study design, exposure protocols, and outcome measures preclude firm conclusions. Similarly, while some animal studies have demonstrated histological alterations in intestinal tissues and changes in gut microbiota composition, these findings require replication and validation in human models.

Importantly, only one human study met the inclusion criteria, highlighting a significant gap in clinical research on NFECs and gastrointestinal health. This paucity of clinical data limits the translational relevance of preclinical findings and underscores the need for carefully designed human studies, particularly longitudinal and interventional research, to assess both acute and chronic impacts. An important limitation of the present review is that only one eligible human pilot study was identified, with the majority of included research being preclinical in nature. While in vitro and animal studies provide valuable mechanistic insights, their findings cannot be directly extrapolated to human health outcomes due to species differences, controlled exposure conditions, and the use of supraphysiological concentrations in some experiments. This translational gap highlights the urgent need for well-designed, adequately powered longitudinal and interventional studies in diverse human populations to assess both the short- and long-term effects of nicotine-free e-cigarettes on gastrointestinal health.

The results of this review also raise broader concerns regarding regulatory oversight and public perception. NFECs are often marketed as harmless alternatives due to the absence of nicotine, despite accumulating evidence that non-nicotine constituents may exert biological effects of their own. The current regulatory landscape, particularly in regions where NFECs fall outside established tobacco control frameworks, may contribute to underestimation of risk among users—especially adolescents and never-smokers. Given the rapid evolution of vaping technologies and formulations, there is an urgent need for regulatory harmonization, safety testing standards, and transparent labelling practices.

There are several limits to this systematic review. There is a lack of cohort studies on human health, preclinical trials, and observational and interventional studies regarding the impact of NFECs on the GIT, as well as a clear differentiation of NC- and NFEC effects. Commonly confounding factors, such as alcohol and traditional tobacco use, diminish adequate quantification and quality of data. In our review, although several studies examined the effects of NFEC liquid exposure, only three specified the PG/VG ratio used in these e-liquid base constituents. The exact constitution of EC liquids is commonly omitted from both consumers and researchers. Currently, only nicotine content is most commonly displayed, and even then, some ECs labelled as NF, particularly non-refillable ECs, may contain nicotine [60]. The lack of regulation and standardization of EC liquid contents, as well as the ability of customization of both heating coils and EC liquids, poses an additional and unprecedented risk to human health [61,62,63]. Additionally, some consumers resort to constructing their own EC liquids, most commonly combining PG and VG in various ratios with the addition of flavours and nicotine on their own terms.

To our knowledge, this is the first systematic review to specifically evaluate the effects of nicotine-free e-cigarette exposure on the gastrointestinal system. The inclusion of both in vitro and in vivo studies allows for a broad yet focused overview of the current evidence. However, several limitations must be acknowledged. The overall number of eligible studies was low, and heterogeneity in experimental designs limited the possibility of meta-analysis. Furthermore, many in vitro studies used supraphysiological concentrations of e-liquids or isolated components, potentially overestimating biological effects. The lack of standardized exposure protocols and variability in cell models and outcome measures also complicates direct comparison between studies.

## 5. Conclusions

This systematic review demonstrates that NFECs are capable of inducing biological effects within the gastrointestinal system, despite the absence of nicotine. The evidence, though limited and primarily preclinical, suggests that components such as propylene glycol, vegetable glycerine, and flavouring agents may contribute to pro-inflammatory, cytotoxic, and microbiota-altering effects, particularly in the oral cavity. Findings from in vitro and in vivo models indicate that NFEC exposure can impair epithelial integrity, alter microbial metabolism, and potentially influence host–microbiome interactions. While data on deeper gastrointestinal segments, including the liver and intestines, remain scarce and heterogeneous, early signals of hepatic metabolic changes and intestinal dysbiosis warrant further investigation.

The prevailing assumption that nicotine-free products are inherently safe is not substantiated by current evidence. Given the increasing popularity of these products, especially among young and previously non-smoking individuals, a more cautious and evidence-based approach to their safety evaluation is essential. Current regulatory frameworks often fail to address the potential risks of NFECs, highlighting a pressing need for policy reassessment and targeted research.

### Future Directions

A key limitation of the current evidence base is the near-complete absence of clinical data, with only one small human pilot study meeting our inclusion criteria. This represents a substantial translational gap, as findings from preclinical models—while critical for understanding underlying mechanisms—cannot be directly extrapolated to real-world human health outcomes due to differences in physiology, exposure patterns, and environmental factors. To address this gap, future research should prioritize well-designed, adequately powered longitudinal and interventional studies in diverse human populations. These studies should examine both acute and chronic gastrointestinal effects of NFEC exposure, use standardized exposure protocols, and assess clinically relevant endpoints such as gastrointestinal symptoms, biomarkers of inflammation, microbial diversity, and epithelial integrity.

In parallel, preclinical models require greater standardization in exposure protocols, substance concentrations, and outcome measures to improve comparability. Mechanistic studies are needed to clarify how e-liquid base constituents (propylene glycol, vegetable glycerine, flavourings) and their thermal degradation products interact with epithelial cells, immune pathways, and the gut microbiome. Given the interconnection of the gastrointestinal system, liver, and central nervous system, research should also address potential effects on the gut–liver–brain axis, with particular attention to microbial dysbiosis and systemic inflammation.

In addition, future studies should adopt a multidisciplinary approach, integrating expertise from toxicology, microbiology, gastroenterology, and public health to ensure a comprehensive evaluation of NFEC-related risks. Continuous monitoring of novel e-cigarette formulations, device technologies, and patterns of use will also be essential to capture emerging hazards and adapt research priorities accordingly.

Finally, research progress should be matched by policy action, including regulatory harmonization, clearer labelling, and transparent communication of potential risks—especially to youth and non-smokers.

## Figures and Tables

**Table 1:** Eligibility criteria.

Inclusion Criteria	Exclusion Criteria
Study type: Observational studies Experimental studies	Study type: Narrative reviews Commentary
Studies evaluating the effects of nicotine-free e-cigarettes on gastrointestinal system	Studies with primary endpoints not related to GIT
Animal and in vitro studies Ex vivo studies Microbiological studies	Research published in languages other than English
	Articles without full-text availability
	Duplicates

**Table 2:** NFEC and oral health.

First Author’s Name/Year/Country	Study Model	Aim	Key Findings	Oral Health Implications
Yu et al./2016 [39]/England	In vitro (human oral keratinocytes and HNSCC cell lines)	To assess genotoxic and cytotoxic potential of NF and NC EC exposure on epithelial and HNSCC cell lines	Similar increases in DNA strand breaks, increased apoptosis, and decreased clonogenic survival in both groups	NFECs may impair gingival tissue repair and promote oral soft tissue damage
Sancilio et al./2016 [40]/Italy	In vitro (primary human gingival fibroblasts)	To determine the cytotoxic and pro-apoptotic effects of EC exposure via ROS production and apoptosis-related protein expression	Both NC and NFEC exposure induced similar increases in ROS production after 24 h, along with increased Bax expression and apoptosis after 48 h of exposure	The cytotoxicity exerted on HGF by EC exposure is not entirely attributable to nicotine
Sancilio et al./2017 [41]/Italy	In vitro (primary human gingival fibroblasts (HFGs))	To examine the structural and molecular changes in fibroblasts exposed to ECs with and without nicotine	Increased production of autophagic vesicles and increased number of pro-collagen I production in fibroblasts exposed to EC	EC exposure may alter tissue remodelling and repair in a nicotine-independent manner
Alanzi et al./2018 [42]/Canada	In vitro (primary human gingival fibroblasts)	To examine how EC exposure with and without nicotine affects human gingival fibroblast migration and wound healing	Decreased cell proliferation rates, delayed wound healing, increased apoptosis in both groups, more pronounced with nicotine	EC exposure impairs tissue repair and regeneration; more pronounced with NC EC exposure
Rouabhia et al./2019 [43]/Canada	In vitro (human osteoblasts on titanium implants)	To assess the effects of TC and EC exposure with and without nicotine on osteoblast interaction with dental implant material	Decreased cell adhesion, ALP activity in osteoblasts, decreased mineralization rates, and increased apoptosis—most pronounced with TC exposure, with comparable effects in NC- and NFEC groups	Osseointegration compromise; dental implant complications minimally attributable to nicotine in TC and EC exposure
Tsai et al./2020 [44]/United States	In vitro study (human gingival and tongue SCC)	To examine the impact of electronic cigarette flavouring and nicotine on SCC and RAGE expression and pro-inflammatory signalling	Increased cell invasion in gingival cells with Red Hot EC flavouring, decreased with Green Apple EC flavour, nicotine-dependent increases in RAGE expression, and differential expression of IL-1 a, IL-8, and MMP	EC flavours may exert a spectrum of effects, including carcinogenic effects
Beklen et al./2021 [45]/England	In vitro (human gingival epithelial cells)	To determine the effects of unflavoured EC exposure on gingival epithelial cells	Decreased cell viability; increased IL-6, IL-8, and MMP-6 production; PG > VG	Inflammatory and cytotoxic potential of main solvents used for transport of nicotine and flavours in EC
Manyanga et al./2021 [46]/United States	In vitro study (human H&N SCC)	Effects of EC exposure on chemotherapeutic resistance of HNSCC	Exposure to EC aerosol increased the concentration of cisplatin needed to induce 50% reduction in cell growth in a nicotine-independent manner	EC exposure may increase chemotherapeutic resistance of HNSCC
Haghighi et al./2022 [47]/United States	In vitro study using *C. albicans* planktonic and biofilm cultures	To investigate and compare effects of pure nicotine, TC, EC, and NFEC exposure on growth, morphology, biofilm formation, and gene expression of *C. albicans*, including interaction with antifungal drugs	Nicotine at low concentrations enhanced *C. albicans* biofilm formation; both TC and EC inhibited growth at much lower nicotine concentrations than pure nicotine; EC stimulates EAP1 and ALS3 expression	Smoking and vaping may enhance *C. albicans* pathogenicity and increased biofilm formation and virulence gene expression; NFEC had minimal effect, suggesting nicotine is the main contributor to virulence enhancements
Tolba et al./2023 [48]/Egypt	In vivo (rat lingual papillae)	Effect of EC exposure via intraperitoneal injections with and without nicotine, as well as pure nicotine, on histomorphological parameters of taste buds and the possibility of damage reversal via vitamin E and C supplementation	Abnormal epithelial stratification and mitotic figs; decrease in taste buds to epithelium surface in all groups	EC exposure may damage taste buds
Ma et al./2024 [49]/United States	In vitro (human oral keratinocytes)	To evaluate cytotoxic and genotoxic effects of EC with and without nicotine	EC aerosols increase cytotoxicity and induce DNA damaging responses	Effects of EC liquids on oral keratinocyte health similar to acrolein treatment
Vamos et al./2024 [50]/Netherlands	Pilot clinical study	How traditional tobacco and electronic cigarettes (NC and NF variants) affect palatal blood flow	Minimal changes in palatal blood flow before and after exposure in any group	Electronic cigarettes may pose acute vascular risk to oral health
Lee-Scott Beverly et al./2025 [51]/United States	In vitro; multi-specimen oral biofilm microcosms + human saliva samples	How e-cigarette aerosols are metabolized by oral microbiome and their effects on microbial metabolism, biofilm architecture, and function	E-cigarette aerosols are metabolized by oral bacteria, altering metabolome and gene expression via xenobiotic degradation and quorum sensing; biofilm density increased, exopolysaccharide-rich, regardless of nicotine content; findings verified in human saliva samples	Oral microbial metabolism of e-cig aerosols may disrupt host–microbiome balance; promote gingival inflammation, caries, antimicrobial resistance; and alter oral mucosal barriers

Legend: EC—e-cigarette; HNSCC—head and neck squamous cell carcinoma; HGF—human gingival fibroblast; NC—nicotine-containing; NF—nicotine-free; NFEC—nicotine-free e-cigarette; SCC—squamous cell carcinoma; MMP—matrix metalloproteinase; PG—propylene glycol; TC—traditional cigarettes; VG—vegetable glycerine.

**Table 3:** NFEC and liver health and function.

First Author’s Name/Year/Country	Study Model	Aim	Key Findings	Hepatic Health Implications
Golli et al./2016 [52]/England	In vivo animal study (adult rat liver)	Effect of ECs on hepatic parameters, lipid peroxidation, and antioxidant activity	Increased LFT probes, reduction in antioxidant enzyme activity, increased lipid peroxidation, inflammatory cell infiltration, and cell death; more pronounced than with nicotine alone	NFEC alters hepatic metabolism and promotes cellular damage; nicotine presence worsens histopathological injuries
Chen et al./2018 [53]/Switzerland	In vivo animal study (mouse dams)	Impact of EC use during pregnancy	Offspring of dams exposed to EC were the heaviest and with the most body fat	Maternal EC exposure may exert long-term metabolic effects in offspring
Li et al./2020 [54]/United States	In vivo (rat offspring with maternal EC exposure during and after gestation)	Effects of EC smoking on hepatic health in dams and their offspring	Metabolic changes and liver damage, notably steatosis; nicotine provides a potential protective effect	Vaping may induce long-term metabolic changes
Rickard et al./2021 [55]/United States	In vitro (HepG2 cells)	Effect of common EC flavours and e-liquid base constituents (PG and VG) on liver cell toxicity	Vanillin, ethyl vanillin, and ethyl maltol decreased HepG2 cell viability; repeated exposure caused increased cytotoxicity	Frequent vaping can cause hepatotoxicity
Chen et al./2021 [56]/Switzerland	In vivo animal study (mice)	Impact of EC exposure with and without nicotine on lipid and glucose profiles as well as liver metabolic markers in mice fed HFD	NFEC exposure increased lipid content in both blood and liver of chow-fed mice	EC exposure may alter hepatic lipid metabolism through adaptive responses
Chen et al./2022 [57]/Switzerland	In vivo animal study (rats)	How NFECs affect inflammatory responses in mice with long-term high-fat diet	Both EC exposure and HFD consumption increased serum IFN-γ, CX3CL1, IL-10, CCL20, CCL12, and CCL5 levels; levels of IFN-γ, CX3CL1, and IL-10 were higher in mice exposed to ECs than those fed on HFD	Short-term NFEC exposure is more potent than long-term HFD consumption in causing systemic inflammation, particularly in liver

Legend: CCL20—C-C motif chemokine ligand 20 (MIP3-α; Macrophage Inflammatory Protein-3 alpha); CCL12—C-C motif chemokine ligand 12 (MCP-5; Monocyte Chemotactic Protein-5); CCL5—C-C motif chemokine ligand 5 (RANTES; Regulated upon Activation, Normal T-cell Expressed and Secreted); CX3CL1—C-X3-C motif chemokine ligand I (Fractalkine); EC—e-cigarette; HepG2—human hepatocellular carcinoma cell line G2; HFD—high-fat diet; LFT—liver function test; IFN-γ—interferon gamma; IL-10—interleukin 10; NFEC—nicotine-free e-cigarette; PG—propylene glycol; VG—vegetable glycerine.

**Table 4:** NFEC and gastrointestinal microbiome.

First Author’s Name/Year/Country	Study Model	Aim	Key Findings	Gut Health Implications
Sharma et al./2021 [58]/United States	In vitro (murine and human enteroid-derived monolayer)	Effects of EC use on the gut barrier	Chronic, but not acute, NFEC exposure increased inflammation and reduced expression of tight junction markers; co-culture with bacteria caused barrier disruption	NFEC liquid components may compromise gut health
Jasper et al./2024 [59]/England	In vitro (human neutrophils)	How EC exposure affects neutrophil function and phenotype	Altered neutrophil surface marker expression, decreased phagocytic function, and excessive filamentous actin polymerization	EC liquids may disrupt neutrophilic function at the gut barrier level

Legend: EC—electronic cigarette; NFEC—nicotine-free e-cigarette.

## Data Availability

No new data were created or analyzed in this study. Data sharing is not applicable to this article.

## Supplementary Materials

The following supporting information can be downloaded at: https://www.mdpi.com/article/10.3390/biomedicines13081998/s1, File S1: Complete search strategies for each database.

## Abbreviations

The following abbreviations are used in this manuscript:

CCL20	C-C motif chemokine ligand 20
CCL12	C-C motif chemokine ligand 12
CCL5	C-C motif chemokine ligand 5
CSC	Cigarette smoke condensate
CX3CL1	C-X3-C motif chemokine ligand I
ECC	E-cigarette smoke condensate
EC	Electronic cigarette, e-cigarette
ENDS	Electronic nicotine delivery system
EU	European Union
EVALI	E-vaping-associated lung injury
FDA	Food and Drug Administration
GIT	Gastrointestinal tract
HFD	High-fat diet
HNSCC	Head and neck squamous cell carcinoma
HGF	Human gingival fibroblast
LFT	Liver function test
IFN-ɣ	Interferon gamma
IL-10	Interleukin-10
MMP	Matrix metalloproteinase
NC	Nicotine-containing
NF	Nicotine-free
NFEC	Nicotine-free e-cigarette
SCC	Squamous cell carcinoma
OSCC	Oral squamous cell carcinoma
PG	Propylene glycol
VG	Vegetable glycerine
