# Supplementary material for: Effects of Nicotine-Free E-Cigarettes on Gastrointestinal System: A Systematic Review
Source: Biomedicines. 2025 Aug 16;13(8):1998. doi: 10.3390/biomedicines13081998 (PMC12383567; doi:10.3390/biomedicines13081998)
Supplement: Supplementary file 1 [file biomedicines-13-01998-s001.zip › biomedicines-3790454-supplementary.pdf]

SUPPLEMENTARY File S1

COCHRANE

„nicotine-free“ AND „e-cigarettes“

„e-cigarettes“

„e-cigarettes“ AND „gut“

„e-cigarettes“ AND „gastrointestinal“

„e-cigarettes“ AND „oral“

„e-cigarettes“ AND „liver“

„e-cigarettes“ AND „microbiome“

MEDLINE

„nicotine-free“ AND „e-cigarettes“ AND „gut“

„nicotine-free“ AND „e-cigarettes“ AND „gastrointestinal“

„nicotine-free“ AND „e-cigarettes“ AND „gut“

„nicotine-free“ AND „e-cigarettes“ AND „liver“

„e-cigarettes“ AND „liver“

„nicotine-free“ AND „e-cigarettes“ AND metabolism

„nicotine-free“ AND „e-cigarettes“ AND „oral“

„nicotine-free“ AND „e-cigarettes“ AND „liver“

„nicotine-free“ AND „e-cigarettes“ AND „microbiome“

„e-cigarettes“ AND „microbiome“

„e-cigarettes“ AND „stomach“

„e-cigarettes“ AND „oral“ AND „microbiome“

„vegetable glycerine“ AND „gastrointestinal“

„propylene glycol“ AND „gastrointestinal“

SCOPUS

„nicotine-free“ AND „e-cigarettes“ AND „gastrointestinal“

„nicotine-free“ AND „e-cigarettes“ AND „stomach“

„e-cigarettes“ AND „oral“

„nicotine-free“ AND „e-cigarettes“ AND „gut“

„nicotine-free“ AND „e-cigarettes“ AND „liver“

„nicotine-free“ AND „e-cigarettes“ AND „oral“

„nicotine-free“ AND „e-cigarettes“ AND metabolism

„e-cigarettes“ AND „gastrointestinal“

„e-cigarettes“ AND „oral“ AND „microbiome“

„e-cigarettes“ AND „oral health“

„nicotine-free“ AND „e-cigarettes“ AND „liver metabolism“

„e-cigarettes“ AND „liver“

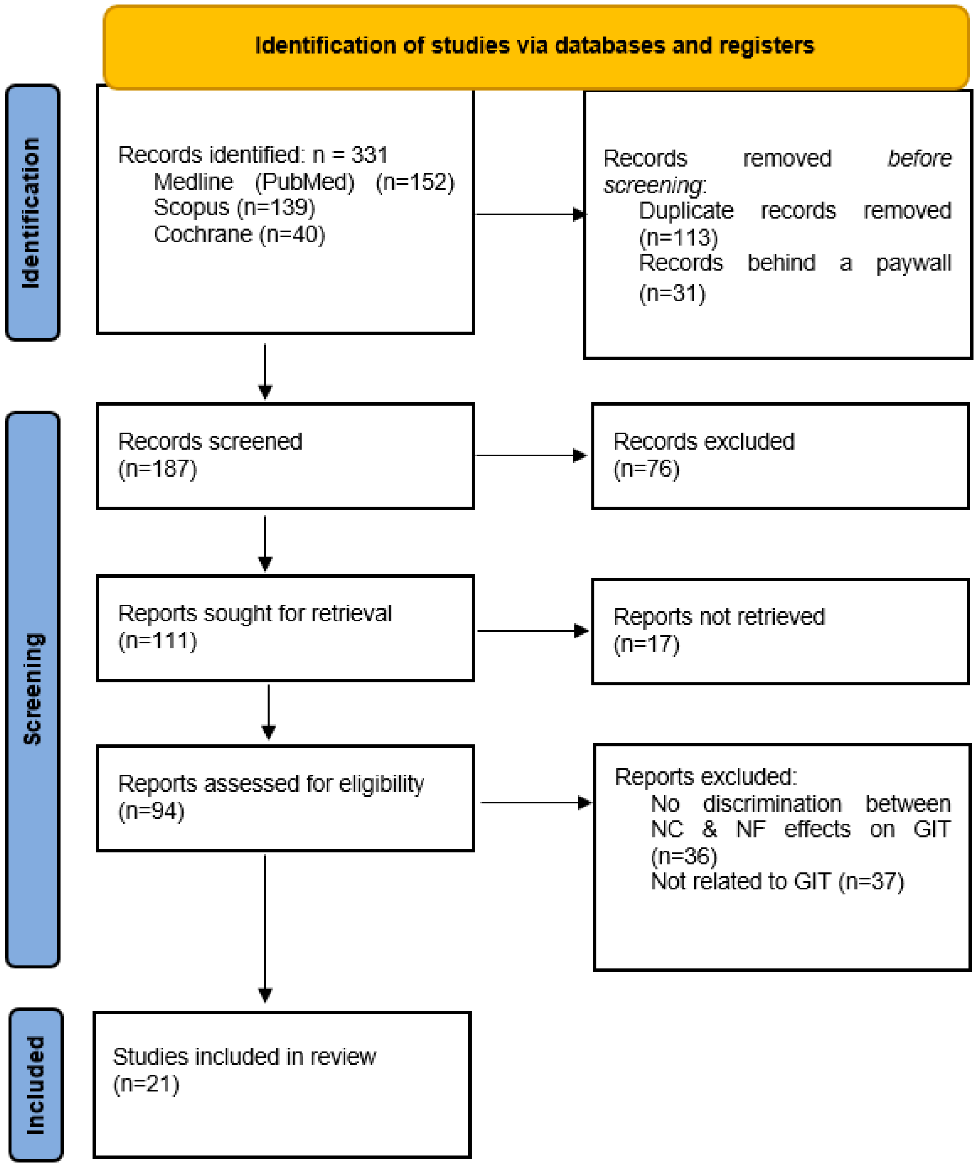

Cochrane – „nicotine-free“ AND „e-cigarettes“ OR „gastrointestinal“ OR „gut“ OR „oral“ or „liver“

Scopus – „nicotine-free e-cigarettes“ AND „gastrointestinal“
